# *Mycobacterium tuberculosis* Complex Genotype Diversity and Drug Resistance Profiles in a Pediatric Population in Mexico

**DOI:** 10.1155/2011/239042

**Published:** 2011-08-23

**Authors:** Mercedes Macías Parra, Jesús Kumate Rodríguez, José Luís Arredondo García, Yolanda López-Vidal, Mauricio Castañón-Arreola, Susana Balandrano, Nalin Rastogi, Pedro Gutiérrez Castrellón

**Affiliations:** ^1^Department of Infectious Diseases, Instituto Nacional de Pediatría, Mexico City, Mexico; ^2^Instituto Mexicano del Seguro Social Foundation, Mexico City, Mexico; ^3^Subdivision of Medical Research, Instituto Nacional de Pediatría, Mexico City, DF, Mexico; ^4^Programa de Inmunología Molecular Microbiana, Departamento de Microbiología y Parasitología, Facultad de Medicina, Universidad Nacional Autónoma de México, Mexico City, Mexico; ^5^Department of Micobacteriology, Instituto De Diagnóstico y Referencia de Epidemiológicos, Secretaria de Salud, Mexico City, Mexico; ^6^WHO Supranational Tuberculosis Reference Laboratory, Institut Pasteur de la Guadeloupe, Abymes, France

## Abstract

The aim of this study was to determine the frequency of drug resistance and the clonality of genotype patterns in *M. tuberculosis* clinical isolates from pediatric patients in Mexico (*n* = 90 patients from 19 states; time period—January 2002 to December 2003). Pulmonary disease was the most frequent clinical manifestation (71%). Children with systemic tuberculosis (TB) were significantly younger compared to patients with localized TB infections (mean 7.7 ± 6.2 years versus 15 ± 3.4 years *P* = 0.001). Resistance to any anti-TB drug was detected in 24/90 (26.7%) of the isolates; 21/90 (23.3%) and 10/90 (11.1%) were resistant to Isoniazid and Rifampicin, respectively, and 10/90 (11.1%) strains were multidrug-resistant (MDR). Spoligotyping produced a total of 55 different patterns; 12/55 corresponded to clustered isolates (*n* = 47, clustering rate of 52.2%), and 43/55 to unclustered isolates (19 patterns were designated as orphan by the SITVIT2 database). Database comparison led to designation of 36 shared types (SITs); 32 SITs (*n* = 65 isolates) matched a preexisting shared type in SITVIT2, whereas 4 SITs (*n* = 6 isolates) were newly created. Lineage classification based on principal genetic groups (PGG) revealed that 10% of the strains belonged to PGG1 (Bovis and Manu lineages). Among PGG2/3 group, the most predominant clade was the Latin-American and Mediterranean (LAM) in 27.8% of isolates, followed by Haarlem and T lineages. The number of single drug-resistant (DR) and multidrug-resistant (MDR-TB) isolates in this study was similar to previously reported in studies from adult population with risk factors. No association between the spoligotype, age, region, or resistance pattern was observed. However, contrary to a study on *M. tuberculosis* spoligotyping in Acapulco city that characterized a single cluster of SIT19 corresponding to the EAI2-Manila lineage in 70 (26%) of patients, not a single SIT19 isolate was found in our pediatric patient population. Neither did we find any shared type belonging to the EAI family which represents ancestral PGG1 strains within the *M. tuberculosis* complex. We conclude that the population structure of pediatric TB in our setting is different from the one prevailing in adult TB patient population of Guerrero.

## 1. Introduction

Although curable, tuberculosis (TB) remains a global public health concern with a major impact in developing countries [1]. Globally, approximately nine million new TB cases and 1.6 million deaths occur every year. The global epidemiology of TB in children is limited; however, the World Health Organization (WHO) estimates that one million cases occurred in children, comprising up to 10% of the total TB incidence [2–4]. Children rarely transmit the disease, and thus contribute little to the maintenance of the TB epidemic even though they make up a substantial proportion of the global disease burden [5]. 

The increasing number of drug-resistant, and especially multidrug-resistant (MDR: defined as resistance to at least isoniazid and rifampicin), cases is a serious concern for the Global TB Control Program instituted by the WHO [6–9]. Treatment of drug-resistant TB is expensive and complex because it necessitates the use of second-line drugs, which are associated with a greater incidence of adverse events [10]. The true extent of drug resistance is unknown, but it is estimated that the global proportion of resistance among all cases is 4.8% (95% confidence interval (CI) 4.6 to 6.0%), with an estimated 489,000 cases of MDR-TB occurring worldwide in 2006. It is thus suggested that resistance to anti-TB drugs may be increasing in some geographical areas, with considerable variation between different countries [11].

Mexico has a declining, but still moderate, incidence of TB that is variable among states (WHO 2005). In 2007, the reported incidence was 13.49 cases/100,000 individuals, with a range of 2.35 to 35.34 cases/100,000 inhabitants and an incidence rate of 5.077/100,000 in children under 19 years of age (Indicadores Demográficos 1990–2030 Consejo CONAPO Sistema Nacional de Vigilancia Epidemiológica, Dirección General de Epidemiología, Secretaria de Salud). Unfortunately, Mexico's surveillance system does not provide an accurate estimation of the drug-resistant strains because the diagnosis of TB is typically performed by direct acid fast bacilli sputum smears. However, limited data from culture-based drug susceptibility testing in 2008 indicate that 2.4% of cases are initially MDR-TB (http://www.cenavece.salud.gob.mx/descargas/pdf/tuberculosis.pdf). There are now up to 479 reported cases of MDR-TB, seven of which were in patients under 19 years of age and 47 that were likely extensively drug-resistant TB (XDR-TB). A strain is considered XDR-TB if it is resistant to isoniazid, rifampicin, any fluoroquinolone and at least one of three injectable second-line drugs (amikacin, kanamycin, or capreomycin); thus, these cases are on the verge of being untreatable [12]. MDR-TB and XDR-TB are among the greatest concerns in the antibiotic resistance pandemic due to the high risk of death [12, 13]. Furthermore, patients can remain infectious for months or even years [14] and may spread MDR- or XDR-TB. Consequently, early detection and an accurate record of individuals the patient has had contact with, are critical in arresting further transmission of the disease and for the proper control of TB.

Study of TB molecular epidemiology through DNA fingerprinting is an important tool contributing to the understanding of the transmission and control of TB [15, 16]. PCR-based spacer oligonucleotide typing (spoligotyping) based on the variability of the direct repeat (DR) locus in the *M. tuberculosis* complex has emerged as a fast, reliable, and cost-effective alternative to the traditional IS*6110* restriction fragment length polymorphism (RFLP) for a first-line genotypic screening of tubercle bacilli [17–19]. More recently, this methodology has been used to provide support for lineage-specific differences and global phylogeography of TB in international databases [20]. In this context, we thought it desirable to focus on pediatric TB since childhood TB is a sentinel event, indicating the ongoing transmission of TB. In addition, these patients are more likely to develop severe cases and extrapulmonary diseases than adults. Unfortunately, there is little information regarding the epidemiology and resistance patterns to anti-TB drugs in children [6]. The aim of this study was to determine the genotype diversity and the extent of drug resistance in *M. tuberculosis* clinical isolates from pediatric patients in Mexico.

## 2. Methods

### 2.1. Patients and Specimens

This study included a total of 90 *Mycobacterium tuberculosis* complex isolates, recruited from patients who were 18 years of age or younger over a 2-year period (January 2002 to December 2003) from 18 federal States in Mexico (see Table  S1 of Supplementary Material available online at doi: 10.1155/2011/239042). Clinical specimen were processes at the local laboratories for culture, followed by identification and drug susceptibility testing (DST) performed at the mycobacteriology laboratory at Instituto De Diagnóstico y Referencia de Epidemiológicos (InDRE) in Mexico city. Isolates were characterized using several biochemical tests: niacin and nitrate positivity; pyrazinamidase activity; negative for catalase activity at 68°C [18]; in-house PCR-RFLP of *hsp65*; determination of antimicrobial resistance using the semiautomated radiometric BACTEC 460TB system according to the manufacturer's instructions (Becton Dickinson, Sparks, MD, USA), with following critical concentrations: streptomycin (STM, 1.0 *μ*g/mL), rifampicin (RMP, 1.0 *μ*g/mL), isoniazid (INH, 0.1 *μ*g/mL), pyrazinamide (PZA, 100 *μ*g/mL), and ethambutol (EMB, 5 *μ*g/mL). Basic demographic data was collected for each patient using a standard questionnaire using the files provided at the local hospitals. The protocol was approved by the Research and Ethics Committee of the Instituto Nacional de Pediatria in Mexico City.

### 2.2. Spoligotyping and Database Comparison

Spoligotyping was performed at the Immunology and Molecular Microbiology Program at the National Autonomous University of Mexico on DNA extracted from heat inactivated cultures using a previously described protocol [19]. Spoligotypes in octal codes were entered in the SITVIT2 proprietary database of the Pasteur Institute of Guadeloupe, which is an updated version of the previously released SpolDB4 database [20]. A Spoligotype International Type (SIT) number was attributed to each pattern according to the SITVIT2 database. At the time of the present study, SITVIT2 contained more than 3000 SITs with global genotyping information on about 75,000 *M. tuberculosis* complex (MTC) clinical isolates from 160 countries of origin. Worldwide distribution of spoligotypes for all clustered isolates (SITs representing 2 or more strains) was investigated using the SITVIT2 database and recorded for countries and regions representing ≥5% of a given SIT as compared to their total number in the global database. The various macrogeographical regions and subregions were defined per UN specifications (http://unstats.un.org/unsd/methods/m49/m49regin.htm). In this database, SIT (Spoligotype International Type) designates spoligotyping shared by 2 or more patient isolates, as opposed to “orphan” which designates patterns reported for a single isolate. Major phylogenetic clades were assigned according to signatures provided in SpolDB4, which defined 62 genetic lineages/sublineages [20]. These include specific signatures for various MTC members such as *M. bovis*, *M. caprae*, *M. microti*, *M. canetti*, *M. pinipedi*, and *M. africanum*, as well as rules defining major lineages/sublineages for *M. tuberculosis sensu stricto*; these include the Beijing clade, the Central Asian (CAS) clade and 2 sublineages, the East African-Indian (EAI) clade and 9 sublineages, the Haarlem (H) clade and 3 sublineages, the Latin American-Mediterranean (LAM) clade and 12 sublineages, the ancestral “Manu” family and 3 sublineages, the S clade, the IS*6110*-low-banding X clade and 3 sublineages, and an ill-defined T clade with 5 sublineages (as well as further well-characterized phylogeographical specificity for 8 additional spoligotype signatures).

### 2.3. Distinction of “Ancient” versus “Modern” Lineages

We also compared the overall repartition of isolates according to major *M. tuberculosis* genotypic families by adding all the shared types for each of the individual family defined (as well as the orphan strains), and further linked the information obtained based on the lineage classification to “ancient” and “modern” lineages of tubercle bacilli as defined by principal genetic groups (PGGs) based on *KatG463-gyrA95* polymorphism [21], inferred from the reported linking of specific spoligotype patterns to PGG1, 2 or 3 [22–24].

### 2.4. Statistical Analyses

SPSS for Windows version 13.0 was used for statistical analyses. Association between categorical variables was assessed by Fisher's exact test and the Mann-Whitney *U* test was used to evaluate associations between numerical variables. Differences between groups were detected by univariate analyses and expressed as the odds ratio (OR) with 95% confidence intervals (95% CI). Differences were considered to be significant if values were <0.05.

## 3. Results 

### 3.1. Study Population

This study included a total of 90 *M. tuberculosis* complex strains isolated from pediatric patients in 18 federal States over a 2-year period (January 2002 to December 2003), which were subsequently referred to the National Institute for Diagnostic and Epidemiological Reference (InDRE) for identification and drug susceptibility testing. These represented between 0.4 and 15.5% of TB cases in this age group as reported by the General Epidemiology Division by those States during the study period. Detailed information on individual strains obtained during this investigation is summarized in Table  S1. Information regarding each strain includes year of isolation, spoligotype description, shared-type number, genotypic lineage, and drug susceptibility to first-line drugs. Additional demographic and clinical information includes pathological specimen, site of infection, geographical origin, age, and sex of the patients.

### 3.2. Clinical Manifestations

*M. tuberculosis* was mostly isolated from sputum samples (77%), mainly in patients with localized TB. In contrast, patients with disseminated disease were diagnosed by spinal fluid (8%), gastric aspirates (4%), urine (2%), pleural fluid (1%), or tissue (8%). Male patients (48) comprised 53% of the population; the mean age of all was 13 ± 5 years, 60% were between 15 and 18 years old and only 8% were under two years of age. Localized TB was diagnosed in 76% of the cases and systemic TB diagnoses corresponded to the remaining 24%. The disease frequently presented as pulmonary TB (65 cases, 71.4%) and was followed by meningeal in 8 cases (8.8%), disseminated in 5 cases (5.5%), miliary in 4 cases (4.4%), abdominal in 3 cases (3.3%), ganglionary in 3 cases (3.3%), urinary in 2 cases (2.2%), and pleural in 1 case (1.1%). Patients with systemic TB were significantly younger (7.7 ± 6.2 years) than children with localized TB (15 ± 3.4 years), *P* = 0.000. Of this population, in 19 of the patients the presence of prior underlying condition was not known. In 10/71 (14%) of the patients, accompanying pathology was identified: 6 (8.4%) were severely malnourished, 2 (2.8%) had a coinfection with human immunodeficiency virus, 1 (1.4%) patient harboring a SIT1/Beijing genotype had diabetes mellitus, and 1 (1.4%) presented with alcoholism. The patients with diabetes mellitus and alcoholism were 18 years of age. The remaining 61 patients had no associated comorbidity.

### 3.3. Drug Resistance

As summarized in Table 1, *M. tuberculosis* isolate showed resistance to at least one anti-TB drug in 24/90 (26.7%) patients; 8 (8.8%) were resistant to a single drug and 4 (4.4%) showed resistance to five first-line TB drugs. The greatest resistance was found against INH (23.3% of cases) followed by RMP (11.1% of cases). Resistance to RMP was always accompanied by INH-resistance leading to 10 (11.1%) cases of MDR-TB. Of 71 patients from whom information was available, 14 patients had previous TB treatment, and secondary resistance was documented in 7/14 (50%) patients in contrast with primary resistance found in 14/57 (24.5%) patients without previous TB treatment (*P* = 0.06).

### 3.4. Risk Factors

Bivariate analysis of risks identified previous treatment as a risk factor for developing resistant to *M. tuberculosis* with an OR = 2.03 (95% CI 1.01 to 4.07, *P* = 0.06). Although this result is not statistically significant, it is of clinical relevance. Furthermore, the risk increased significantly in MDR-TB cases with an OR = 5.08 (95% CI 1.5 to 16.5, *P* = 0.001). We did not find significant differences between age and the presence or not of drug-resistant *M. tuberculosis* (resistant 13.7 ± 5.4 years versus non resistant 13.1 ± 5.2 years, *P* = 0.4). Nevertheless, age was a significant factor (*P* = 0.005) in patients with MDR-TB (16 ± 2.2 years) compared to patients with drug-sensitive TB (12.9 ± 5.4 years). Drug-resistant isolates were identified in 27.5% of patients with both localized and disseminated disease (*P* = 0.44). Patients with MDR-TB were less likely to present systemic TB although this was not statistically significant, (localized 13% versus systemic 4.5%, *P* = 0.24). The prevalence of drug-resistant strains showed variation between different Mexican states. In seven of the twelve states where drug resistance was documented, MDR-TB strains were identified. Follow-up treatment (6 months for pulmonary TB and 12 months for other clinical forms) was documented in 65 (71%) patients; 6 of these patients (9%) died, 2 (3%) stopped treatment, and 1 (1%) patient reported treatment failure. In this group, the presence of drug resistance or MDR did not contribute to the risk of death.

### 3.5. Spoligotyping

Spoligotyping produced a total of 55 different patterns for the 90 isolates studied. The succinct analyses obtained of the prevailing genotypes within this subpopulation as compared to the spoligotypes recorded in the SITVIT2 database are summarized in Tables 2, 3, and 4. Nineteen patterns corresponded to orphan strains that were unique among all the strains recorded in the SITVIT2 database (Table 2), as opposed to 36 patterns from 71 patients that corresponded to shared types (Table 3), that is, an identical pattern shared by 2 or more patients worldwide (within this study, or matching another strain in the SITVIT2 database). As shown in Table 3 for the 36 SITs recorded, a total of 32 SITs (containing 65 isolates) matched a preexisting shared type in the SITVIT2 database, whereas 4 SITs (containing 6 isolates) were newly created either within the present study or after a match with an orphan in the database. Irrespective of the database comparison, 12 patterns corresponded to clusters in the present study (Table 4, 12 clusters containing 47 isolates, 2–12 isolates per cluster), accounting for a clustering rate of 52.2% (47/90). However, no statistically significant association between spoligotype patterns, age, region, drug resistance, or HIV serology was observed.

### 3.6. Distinction of “Ancient” versus “Modern” Lineages

Spoligotyping results and clade definitions were linked to the distribution of clinical isolates within the principal genetic group PGG1 versus PGG2/3 (the latter being putatively characterized by the lack of spacers 33–36), it was evident that only 10/90 (11.1%) strains belonged to PGG1 (BOVIS, Manu and Beijing/W lineages), the rest being classified as evolutionary recent PGG2/3 strains. Indeed, LAM was the most predominant clade (25/90 or 27.8% of the isolates, including the major cluster composed of SIT42/LAM9 sublineage, *n* = 12/90 or 13.3% of the isolates), followed by the Haarlem and T families.

## 4. Discussion

Our study aimed to estimate the genotype diversity and the global prevalence of drug-resistant TB in pediatric patient population. This is the first report of resistance to primary antimicrobial drugs in an exclusively pediatric Mexican population. The extent of childhood TB is unknown and is estimated that has a limited impact on the dynamics of the TB epidemic; however, pediatric TB indicates recent transmission of a disease that has not been previously treated or diagnosed. Therefore, childhood TB can be a reflection on the inefficiency of programs for the control of the disease. In this study, a high frequency of primary antimicrobial resistance was observed (1 out of every 4 isolates). Resistance to INH alone or in combination with other anti-TB drugs was most prevalent, with a global resistance of 23%, a finding which is higher than the 18% prevalence previously reported in population based studies in Mexico [25, 26]. In this respect, our observation is similar to prevalence of 29% to 56% reported in studies in Chiapas and Veracruz (Mexico) for high-risk populations [27–29]. When the prevalence of INH resistance is ≥4%, the use of a fourth drug (myambutol or streptomycin) is recommended to ensure therapeutic success; a standard that was adopted in Mexico in 2000 as part of the TAES strategy—Norma oficial Mexicana NOM-006-SSA2-1993 para la prevención y control de la tuberculosis para la atención primaria de la salud (http://www.salud.gob.mx/unidades/cdi/nom/m006ssa23.html). 

Importantly, the magnitude of RMP-resistance in our study (11%) is higher than previously determined level in Mexico (3%). Resistance to RMP was always accompanied by INH-resistance leading to 10 (11.1%) cases of MDR-TB. Although because of the small number of isolates studied, this high rate of MDR-TB observed (11%) might not reflect the exact rates of drug resistance in this age group, this finding is relevant because RMP and INH are among the most important 1st-line drugs involved both in the TAES strategy (see above) as well as in the WHO-recommended Directly Observed Therapy—Short Course (DOTS) strategy (http://www.who.int/tb/dots/en/index.html). MDR-TB is linked to a greater probability of therapeutic failure and a lower survival rate, with a calculated relative risk for mortality of 2.5 (95% CI 1.02 to 6.16, **P** = 0.04) [29]. The occurrence of MDR-TB has logistical implications in the necessity of tests for drug resistance and the use of second-line drugs. 

Because of the limited number of patients in our study, it is difficult to compare the mortality of MDR-TB, which is lower than reported in other national studies [30, 31]. Distribution clusters of any kind were not observed for resistant *M. tuberculosis* isolates, however, 20% of patients were retreated due to therapeutic failure in our study, a finding which is in agreement with previous reports showing that previous TB treatment is a predictor for resistance [32, 33]. These observations stress the importance of performing periodical population studies to determine the prevalence and incidence of drug-resistant *M. tuberculosis* and to establish the most effective strategies for the control TB. 

Spoligotyping of the clinical isolates showed the great diversity of circulating genotypes, with 55 different patterns which is common in countries with endemic. The spoligotypes were not associated with the geographical location of the patient or the presence of resistance to one or more antimicrobial drugs. The worldwide distribution of predominant shared types and lineages from our study underlined that many of the genotypes encountered in the pediatric population were the ones most frequently represented in Mexico and neighboring USA, the rest being essentially shared with other Latin American and Caribbean neighbors (Table 4). Thus with the exception of ubiquitous spoligotypes (such as the T clade found throughout the world), the patients in our study mainly harboured *M. tuberculosis* spoligotypes prevailing in the Americas, and mostly belonging to evolutionary recent PGG2/3 lineages. However, the “T” genotype does not represent a clade in a strict evolutionary sense since it was defined by default to include strains that may not be classified in one of the established genotypic lineages with well-established phylogeographical specificity such as the Haarlem, LAM, CAS, and EAI lineages [20]. Interestingly, out of the 12 sublineages reported worldwide for the LAM clade [20], a total of 6 sublineages were present in our 2-year recruitment. Last but not least, the near absence of Beijing genotype (**n** = 1) in this study is noteworthy and none were associated with 2 HIV patients (one of the HIV patients harbored a T1 lineage strain). 

A remarkable feature of our study is the presence of a few ancestral Manu lineage strains (**n** = 4/90 or 4.4%) as clustered strains within our study sample. The “Manu” lineage was initially described as a new family from India in 2004 [34], and later similar strains in small numbers were reported in a study from Madagascar [35]. Soon afterwards, it was tentatively subdivided into Manu-1 (deletion of spacer 34), Manu-2 (deletion of spacers 33-34), and Manu-3 (deletion of spacers 34–36) sublineages, and it was suggested that it could represent an ancestral clone of principal genetic group 1 strains [20]. Manu lineage strains were recently reported from Saudi Arabia [36], Tunisia [37], and more recently in a study from Egypt where it represented as high as 27% of all isolates [38]. 

Contrary to a recent study on *M. tuberculosis* spoligotyping in Acapulco city that showed a single cluster of 70 (26%) patients harboring a single spoligotype (SIT19) corresponding to the EAI2-Manila lineage [39], we did not find a single SIT19 isolate in the pediatric patient population of our study. Neither did we find any shared type belonging to the EAI family which represents ancestral PGG1 strains within the *M. tuberculosis* complex [20]. We therefore conclude that the population structure of pediatric TB in our setting is entirely different from the one prevailing in adult TB patient population of Guerrero with evidence of an ongoing transmission with ancestral EAI2-Manila lineage [39]. In conclusion, our study shows that TB among pediatric patients in Mexico is essentially caused by evolutionary recent genotypes characteristic of the Americas. However, the presence of the ancestral Manu lineage strains, supposed to be a missing link of the split between ancestral and modern tubercle bacilli during *M. tuberculosis* evolution [38], should be further investigated within larger datasets to know its arrival in Mexico. Lastly, the clustering rate observed in our study (52.2%) is certainly higher than expected since further differentiation of spoligotyping defined clusters was not systematically performed using secondary markers such as MIRU-VNTRs [40], which will be necessary in future studies to draw epidemiological conclusions. 

## Supplementary Material

Table S1 shows detailed information on individual *M. tuberculosis* complex clinical isolates from Mexican pediatric patients. It includes: year of isolation, spoligotype description, shared-type number, genotypic lineage, and drug-susceptibility to first-line drugs. Additional demographic and clinical information includes: pathological specimen, site of infection, geographical origin, age and sex of the patients.

## Figures and Tables

**Table 1:** Characteristics of *M. tuberculosis* drug resistance to first line antituberculosis drugs.

Number of drugs	Number of isolates	Resistance to antituberculosis drugs (no and %)				
		INH	STM	RMP	PZA	EMB
1	8	5 (23.8)	3 (21.2)	—	—	—
2	6	6 (28.5)	2 (18.1)	2 (20.0)	1 (12.5)	1 (11.1)
3	5	5 (23.8)	2 (18.1)	3 (30.0)	2 (25.0)	3 (33.3)
4	1	1 (4.7)	—	1 (10.0)	1 (12.5)	1 (11.1)
5	4	4 (19.0)	4 (36.3)	4 (40.0)	4 (50.0)	4 (44.1)
Global resistance	24	21 (23.3)	11 (12.2)	10 (11.1)	8 (8.8)	9 (10.0)

INH: Isoniazid, STM: Streptomycin, RMP: Rifampicin, PZA: Pyrazinamide, EMB: Ethambutol.

**Table 2:** Description of orphan strains (*n* = 19) and corresponding spoligotyping defined lineages.

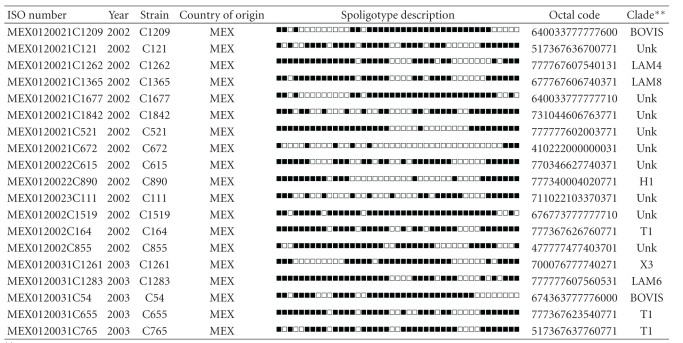

**Clade designations are according to SITVIT2 database. Unk: unknown patterns within major clades described in SITVIT2.

**Table 3:** Description of *M. tuberculosis* complex shared types in pediatric patients (*n* = 90).

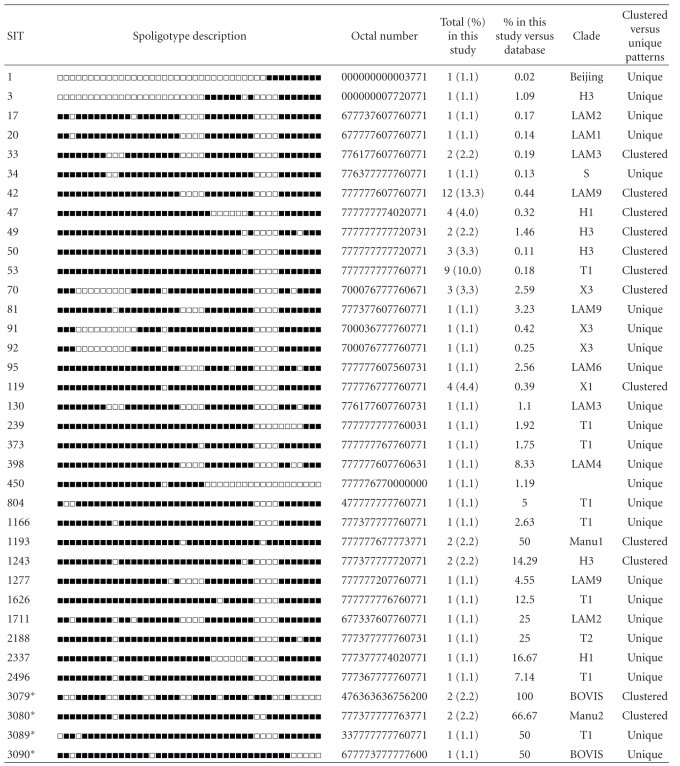

Shared types (SIT) followed by an asterisk indicate “newly created” shared types (*n* = 4) due to 2 or more strains belonging to an identical new pattern within this study (SIT3079, SIT3080), or a unique strain from this study matching with another orphan in the database (SIT3089 matched an orphan from Peru; SIT3090 matched an orphan from USA). Note that SIT3080 was created by 2 strains belonging to an identical pattern within this study that also matched an orphan from Saudi Arabia.

Clade designations are according to SITVIT2 database. Unk: unknown patterns within major clades described in SITVIT2.

Clustered versus unique patterns; clustered strains correspond to a similar spoligotype pattern shared by 2 or more strains “within this study”; as opposed to unique strains harboring a spoligotype pattern that does not match with another strain from this study. Unique strains matching a preexisting pattern in the SITVIT2 database are classified as SITs, whereas in case of no match, they are designated as “orphan” (see Table 1).

**Table 4:** Description of shared types representing clustered strains and their worldwide distribution.

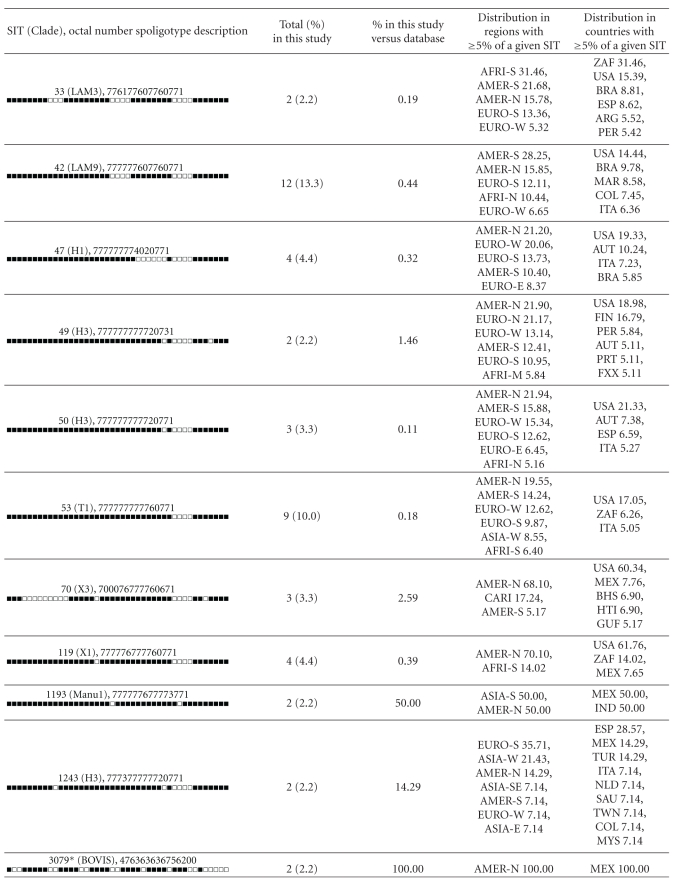 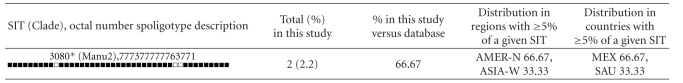

Worldwide distribution is reported for regions with ≥5% of a given SITs as compared to their total number in the SITVIT2 database. The definition of macrogeographical regions and subregions (http://unstats.un.org/unsd/methods/m49/m49regin.htm) is according to the United Nations; Regions: AFRI (Africa), AMER (Americas), ASIA (Asia), EURO (Europe), and OCE (Oceania), subdivided in: E (Eastern), M (Middle), C (Central), N (Northern), S (Southern), SE (South-Eastern), and W (Western). Furthermore, CARIB (Caribbean) belongs to Americas, while Oceania is subdivided in 4 subregions: AUST (Australasia), MEL (Melanesia), MIC (Micronesia), and POLY (Polynesia). Note that in our classification scheme, Russia has been attributed a new sub-region by itself (Northern Asia) instead of including it among rest of the Eastern Europe. It reflects its geographical localization as well as due to the similarity of specific TB genotypes circulating in Russia (a majority of Beijing genotypes) with those prevalent in Central, Eastern, and South-Eastern Asia.

The 3 letter country codes are according to http://en.wikipedia.org/wiki/ISO_3166-1_alpha-3; countrywide distribution is only shown for SITs with ≥5% of a given SITs as compared to their total number in the SITVIT2 database.
