# Supplementary material for: Mycobacterium tuberculosis Complex Genotype Diversity and Drug Resistance Profiles in a Pediatric Population in Mexico
Source: Tuberc Res Treat. 2011 Aug 23;2011:239042. doi: 10.1155/2011/239042 (PMC3335619; doi:10.1155/2011/239042)
Supplement: Supplementary file 1 — Table S1 shows detailed information on individual M. tuberculosis complex clinical isolates from Mexican pediatric patients. It includes: year of isolation, spoligotype description, shared-type number, genotypic lineage, and drug-susceptibility to first-line drugs. Additional demographic and clinical information includes: pathological specimen, site of infection, geographical origin, age and sex of the patients. [file 239042.f1.pdf]

SUPPLEMENTAL TABLE S1: Detailed information on M. tuberculosis complex clinical isolates from Mexican pediatric patients (n=90).

Year	Strain	Spoligotype Description	Octal code	Genotypic Lineage	SIT*	Drug susceptibility**	Clinical specimen	Site of infection	Geographical origin	Sex/Age
2003	C1277	□□□□□□□□□□□□□□□□□□□□□□□□□□□□□□□□□□■■■■■■■■■	000000000003771	Beijing	1	Pansusceptible	sputum	Pulmonary	Jalisco	M/ 16 yrs
2002	C1472	□□□□□□□□□□□□□□□□□□□□□□□□■■■■■■□■□□□□■■■■■■■	000000007720771	H3	3	Pansusceptible	sputum	Pulmonary	Jalisco	F/12 yrs
2002	C892	■■□■■■■■■■■■□■■■■■■■□□□□■■■■■■■■□□□□■■■■■■■	677737607760771	LAM2	17	Pansusceptible	sputum	Pulmonary	Jalisco	F/ 18 yrs
2002	C1417	■■□■■■■■■■■■■■■■■■■□□□□■■■■■■■■□□□□■■■■■■■	677777607760771	LAM1	20	Pansusceptible	sputum	Pulmonary	Hidalgo	F/14 yrs
2002	C374	■■■■■■■■□□□■■■■■■■■■□□□□■■■■■■■■□□□□■■■■■■■	776177607760771	LAM3	33	Pansusceptible	sputum	Pulmonary	Jalisco	M/ 16 yrs
2002	C663	■■■■■■■■□□□■■■■■■■■■□□□□■■■■■■■■□□□□■■■■■■■	776177607760771	LAM3	33	Resistant= I	sputum	Pulmonary	Sinaloa	M/ 14 yrs
2002	C1735	■■■■■■■■□□■■■■■■■■■■■■■■■■■■■■■■□□□□■■■■■■■	776377777760771	S	34	Pansusceptible	CSF	Extra Pulmonary (Meninges)	Tabasco	M/ 7 yrs
2002	C1379	■■■■■■■■■■■■■■■■■■■■□□□□■■■■■■■■□□□□■■■■■■■	777777607760771	LAM9	42	Pansusceptible	sputum	Pulmonary	Tamaulipas	M/18 yrs
2002	C1740	■■■■■■■■■■■■■■■■■■■■□□□□■■■■■■■■□□□□■■■■■■■	777777607760771	LAM9	42	Pansusceptible	sputum	Pulmonary	Sinaloa	M/ 16 yrs
2002	C1786	■■■■■■■■■■■■■■■■■■■■□□□□■■■■■■■■□□□□■■■■■■■	777777607760771	LAM9	42	Pansusceptible	gastric lavage	Extra Pulmonary (Systemic)	D. F	F/9 months
2002	C330	■■■■■■■■■■■■■■■■■■■■□□□□■■■■■■■■□□□□■■■■■■■	777777607760771	LAM9	42	Pansusceptible	sputum	Extra Pulmonary (Milliar)	Edo de México	M/ 8 yrs
2002	C9	■■■■■■■■■■■■■■■■■■■■□□□□■■■■■■■■□□□□■■■■■■■	777777607760771	LAM9	42	Resistant= S, I, Rif, Et, Pz	sputum	Pulmonary	Guanajuato	F/ 17 yrs
2002	C1438	■■■■■■■■■■■■■■■■■■■■□□□□■■■■■■■■□□□□■■■■■■■	777777607760771	LAM9	42	Resistant= I	sputum	Pulmonary	Sinaloa	M/ 16 yrs
2002	C836	■■■■■■■■■■■■■■■■■■■■□□□□■■■■■■■■□□□□■■■■■■■	777777607760771	LAM9	42	Resistant= I	sputum	Pulmonary	Chiapas	F/ 12 yrs
2003	C1021	■■■■■■■■■■■■■■■■■■■■□□□□■■■■■■■■□□□□■■■■■■■	777777607760771	LAM9	42	Pansusceptible	CSF	Extra Pulmonary (Meninges)	Guerrero	M/ 7 yrs
2003	C861	■■■■■■■■■■■■■■■■■■■■□□□□■■■■■■■■□□□□■■■■■■■	777777607760771	LAM9	42	Pansusceptible	sputum	Pulmonary	Tamaulipas	M/ 18 yrs
2003	C269a	■■■■■■■■■■■■■■■■■■■■□□□□■■■■■■■■□□□□■■■■■■■	777777607760771	LAM9	42	Resistant= S, I, Rif, Et, Pz	sputum	Pulmonary	Guerrero	M/ 17 yrs
2003	C1194	■■■■■■■■■■■■■■■■■■■■□□□□■■■■■■■■□□□□■■■■■■■	777777607760771	LAM9	42	Resistant= S	sputum	Pulmonary	Jalisco	F/ 14 yrs
2003	C885	■■■■■■■■■■■■■■■■■■■■□□□□■■■■■■■■□□□□■■■■■■■	777777607760771	LAM9	42	Resistant= I	sputum	Pulmonary	Jalisco	M/ 14 yrs
2002	C1154	■■■■■■■■■■■■■■■■■■■■■■■■■□□□□□□■□□□□■■■■■■■	777777774020771	H1	47	Pansusceptible	sputum	Pulmonary	Guanajuato	F/ 18 yrs
2002	C131	■■■■■■■■■■■■■■■■■■■■■■■■■□□□□□□■□□□□■■■■■■■	777777774020771	H1	47	Pansusceptible	sputum	Pulmonary	Guanajuato	F/ 11 yrs
2002	C1300	■■■■■■■■■■■■■■■■■■■■■■■■■□□□□□□■□□□□■■■■■■■	777777774020771	H1	47	Resistant= S, I, Rif, Et, Pz	sputum	Extra Pulmonary (Milliary)	Baja California	F/ 15 yrs
2003	C56	■■■■■■■■■■■■■■■■■■■■■■■■■□□□□□□■□□□□■■■■■■■	777777774020771	H1	47	Pansusceptible	CSF	Extra Pulmonary (Meninges)	Sinaloa	M/ 15 yrs
2003	C158	■■■■■■■■■■■■■■■■■■■■■■■■■■■■■■□■□□□□■■■□■■■	777777777720731	H3	49	Pansusceptible	biopsy	Extra Pulmonary (Lymph nodules)	Sinaloa	F/ 5 yrs
2003	C417	■■■■■■■■■■■■■■■■■■■■■■■■■■■■■■□■□□□□■■■□■■■	777777777720731	H3	49	Pansusceptible	biopsy	Extra Pulmonary (Lymph nodules)	Edo De México	M/ 6 yrs
2002	C101	■■■■■■■■■■■■■■■■■■■■■■■■■■■■■■□■□□□□■■■■■■■	777777777720771	H3	50	Pansusceptible	sputum	Pulmonary	Baja California	M/ 11 yrs
2003	C1013	■■■■■■■■■■■■■■■■■■■■■■■■■■■■■■□■□□□□■■■■■■■	777777777720771	H3	50	Resistant= I, R= Rif	sputum	Pulmonary	Colima	M/ 18 yrs
2003	C510	■■■■■■■■■■■■■■■■■■■■■■■■■■■■■■□■□□□□■■■■■■■	777777777720771	H3	50	Resistant= I, Rif, Et	sputum	Pulmonary	Puebla	M/ 18 yrs
2002	C102	■■■■■■■■■■■■■■■■■■■■■■■■■■■■■■■■□□□□■■■■■■■	777777777760771	T1	53	Pansusceptible	sputum	Pulmonary	Baja California	M/ 16 yrs
2002	C1595	■■■■■■■■■■■■■■■■■■■■■■■■■■■■■■■■□□□□■■■■■■■	777777777760771	T1	53	Pansusceptible	sputum	Extra Pulmonary (Abdominal)	Oaxaca	M/ 18 yrs
2002	C1631	■■■■■■■■■■■■■■■■■■■■■■■■■■■■■■■■□□□□■■■■■■■	777777777760771	T1	53	Pansusceptible	sputum	Pulmonary	Sinaloa	F/ 17 yrs
2002	C28	■■■■■■■■■■■■■■■■■■■■■■■■■■■■■■■■□□□□■■■■■■■	777777777760771	T1	53	Pansusceptible	sputum	Pulmonary	Guanajuato	F/ 17 yrs
2002	C514	■■■■■■■■■■■■■■■■■■■■■■■■■■■■■■■■□□□□■■■■■■■	777777777760771	T1	53	Pansusceptible	sputum	Pulmonary	Jalisco	M/ 16 yrs
2002	C996	■■■■■■■■■■■■■■■■■■■■■■■■■■■■■■■■□□□□■■■■■■■	777777777760771	T1	53	Pansusceptible	sputum	Pulmonary	Baja California	F/ 15 yrs
2003	C41	■■■■■■■■■■■■■■■■■■■■■■■■■■■■■■■■□□□□■■■■■■■	777777777760771	T1	53	Pansusceptible	sputum	Pulmonary	Jalisco	M/ 18 yrs
2003	C69	■■■■■■■■■■■■■■■■■■■■■■■■■■■■■■■■□□□□■■■■■■■	777777777760771	T1	53	Pansusceptible	sputum	Pulmonary	Guerrero	M/ 11 yrs
2003	C77	■■■■■■■■■■■■■■■■■■■■■■■■■■■■■■■■□□□□■■■■■■■	777777777760771	T1	53	Pansusceptible	sputum	Pulmonary	Guanajuato	F/ 18 yrs
2002	C924	■■■□□□□□□□□□■■■■■□■■■■■■■■■■■■■■□□□□■■□■■■■	700076777760671	X3	70	Pansusceptible	sputum	Pulmonary	Guanajuato	F/ 16 yrs
2002	C654	■■■□□□□□□□□□■■■■■□■■■■■■■■■■■■■■□□□□■■□■■■■	700076777760671	X3	70	Resistant= S, I, Rif, Et, Pz	sputum	Pulmonary	Guanajuato	F/ 18 yrs
2003	C790	■■■□□□□□□□□□■■■■■□■■■■■■■■■■■■■■□□□□■■□■■■■	700076777760671	X3	70	Pansusceptible	sputum	Pulmonary	Baja California	M/ 18 yrs
2002	C899	■■■■■■■■■□■■■■■■■■■■□□□□■■■■■■■■□□□□■■■■■■■	777377607760771	LAM9	81	Pansusceptible	urine	Extra Pulmonary (Urinary)	Jalisco	F/13 yrs
2002	C752	■■■□□□□□□□□□□■■■■□■■■■■■■■■■■■■■□□□□■■■■■■■	700036777760771	X3	91	Pansusceptible	CSF	Extra Pulmonary (Meninges)	Baja California	M/2 yrs
2002	C1319	■■■□□□□□□□□□■■■■■□■■■■■■■■■■■■■■□□□□■■■■■■■	700076777760771	X3	92	Resistant= S, Resistant=I	urine	Extra Pulmonary (Urinary)	Tamaulipas	F/16 yrs
2002	C345	■■■■■■■■■■■■■■■■■■■■□□□□■■■■□■■■□□□□■■■□■■■	777777607560731	LAM6	95	Pansusceptible	sputum	Pulmonary	Nayarit	M/14 yrs
2002	C814	■■■■■■■■■■■■■■■■■□■■■■■■■■■■■■■■□□□□■■■■■■■	777776777760771	X1	119	Pansusceptible	sputum	Pulmonary	Tamaulipas	M/ 12 yrs
2002	C235	■■■■■■■■■■■■■■■■■□■■■■■■■■■■■■■■□□□□■■■■■■■	777776777760771	X1	119	Resistant= I, Rif, Pz	sputum	Pulmonary	Jalisco	F/ 17 yrs
2003	C753	■■■■■■■■■■■■■■■■■□■■■■■■■■■■■■■■□□□□■■■■■■■	777776777760771	X1	119	Pansusceptible	sputum	Pulmonary	Yucatán	F/ 18 yrs
2003	C823	■■■■■■■■■■■■■■■■■□■■■■■■■■■■■■■■□□□□■■■■■■■	777776777760771	X1	119	Pansusceptible	sputum	Pulmonary	Sinaloa	F/ 18 yrs
2003	C1121	■■■■■■■■□□□■■■■■■■■■□□□□■■■■■■■■□□□□■■■□■■■	776177607760731	LAM3	130	Pansusceptible	sputum	Pulmonary	Baja California	F/ 17 yrs
2003	C269b	■■■■■■■■■■■■■■■■■■■■■■■■■■■■■■■■□□□□□□□□■■■	777777777760031	T1	239	Resistant= S, I, Rif, Et, PZ	sputum	Pulmonary	Guerrero	M/ 17 yrs
2002	C1321	■■■■■■■■■■■■■■■■■■■■■■■□■■■■■■■■□□□□■■■■■■■	777777767760771	T1	373	Pansusceptible	sputum	Pulmonary	Tamaulipas	F/16 yrs
2002	C30	■■■■■■■■■■■■■■■■■■■■□□□□■■■■■■■■□□□□■■□□■■■	777777607760631	LAM4	398	Resistant= S, I, Et	sputum	Pulmonary	Guanajuato	M/18 yrs
2003	C43	■■■■■■■■■■■■■■■■■□■■■■■■□□□□□□□□□□□□□□□□□□□	777776770000000		450	Pansusceptible	CSF	Extra Pulmonary (Meninges)	D F	M/ 18 yrs
2002	C372	■□□■■■■■■■■■■■■■■■■■■■■■■■■■■■■■■□□□□■■■■■■■	477777777760771	T1	804	Resistant= S	sputum	Pulmonary	Jalisco	M/18 yrs
2002	C1531	■■■■■■□■■■■■■■■■■■■■■■■■■■■■■■■■□□□□■■■■■■■	773777777760771	T1	1166	Pansusceptible	sputum	Extra Pulmonary (Abdominal)	Edo de México	M/7 yrs
2002	C327	■■■■■■■■■■■■■■■■■■■■□■■■■■■■■■■■■■□■■■■■■■■■	777777677773771	MANU1	1193	Pansusceptible	sputum	Pulmonary	Edo de México	M/ 7 yrs
2002	C670	■■■■■■■■■■■■■■■■■■■■□■■■■■■■■■■■■■□■■■■■■■■■	777777677773771	MANU1	1193	Pansusceptible	biopsy	Extra Pulmonary (Systemic)	Yucatán	M/ 1 yrs
2002	C1473	■■■■■■□■■■■■■■■■■■■■■■■■■■■■■■□■□□□□■■■■■■■	773777777720771	H3	1243	Pansusceptible	sputum	Pulmonary	Jalisco	M/ 18 yrs
2002	C1783	■■■■■■□■■■■■■■■■■■■■■■■■■■■■■■□■□□□□■■■■■■■	773777777720771	H3	1243	Resistant= I, Et	gastric lavage	Extra Pulmonary (Systemic)	D F	F/ 9 months
2003	C665	■■■■■■■■■■■■■■■■■■□■□□□□■■■■■■■■□□□□■■■■■■■	777777207760771	LAM9	1277	Pansusceptible	CSF	Extra Pulmonary (Meninges)	Tabasco	F/ 8 yrs
2003	C1232	■■■■■■■■■■■■■■■■■■■■■■■■■■□■■■■■□□□□■■■■■■■	777777776760771	T1	1626	Pansusceptible	biopsy	Extra Pulmonary (Systemic)	Sinaloa	M/ 2 yrs

2002	C37	■■□■■■■■■□■■□■■■■■■■□□□□■■■■■■■■□□□□■■■■■■■	677337607760771	LAM2	1711	Pansusceptible	sputum	Pulmonary	Jalisco	M/ 17 yrs
2002	C85	■■■■■■■■■□■■■■■■■■■■■■■■■■■■■■■■□□□□■■■□■■■	777377777760731	T2	2188	Pansusceptible	sputum	Pulmonary	Oaxaca	M/15 yrs
2002	C1692	■■■■■■■■■□■■■■■■■■■■■■■■■□□□□□□■□□□□■■■■■■■	777377774020771	H1	2337	Pansusceptible	sputum	Pulmonary	Baja California	M/18 yrs
2003	C870	■■■■■■■■■□■■■■□■■■■■■■■■■■■■■■■■□□□□■■■■■■■	777367777760771	T1	2496	Pansusceptible	sputum	Pulmonary	Hidalgo	F/13 yrs
2003	C1011	■□□■■■■■□□■■■■□□■■■■□□■■■■□■■■■□■■■□□■□□□□□	476363636756200	BOVIS	3079*	Pansusceptible	sputum	Pulmonary	Tamaulipas	M/ 17 yrs
2003	C949	■□□■■■■■□□■■■■□□■■■■□□■■■■□■■■■□■■■□□■□□□□□	476363636756200	BOVIS	3079*	Pansusceptible	sputum	Pulmonary	Jalisco	F/17 yrs
2002	C1742	■■■■■■■■■□■■■■■■■■■■■■■■■■■■■■■■□□■■■■■■■■■	777377777763771	MANU2	3080*	Pansusceptible	sputum	Pulmonary	Sinaloa	F/ 17 yrs
2002	C1853	■■■■■■■■■□■■■■■■■■■■■■■■■■■■■■■■□□■■■■■■■■■	777377777763771	MANU2	3080*	Pansusceptible	CSF	Extra Pulmonary (Meninges)	Jalisco	M/5 yrs
2002	C1785	□■■□■■■■■■■■■■■■■■■■■■■■■■■■■■■■□□□□■■■■■■■	337777777760771	T1	3089*	Pansusceptible	gastric lavage	Pulmonary	Oaxaca	F/16 yrs
2002	C998	■■□■■■■■■■■■■■■□■■■■■■■■■■■■■■■■■■■■■■□□□□□	677773777777600	BOVIS	3090*	Pansusceptible	sputum	Pulmonary	Baja California	M/15 yrs
2002	C1209	■■□■□□□□□□□□□■■□■■■■■■■■■■■■■■■■■■■■■■□□□□□	640033777777600	BOVIS	Orphan	Pansusceptible	sputum	Pulmonary	Jalisco	M/17 yrs
2002	C121	■□■□□■■■■□■■■■□■■■■■□□■■■■□■■■□□□□□□■■■■■■■	517367636700771		Orphan	Pansusceptible	sputum	Pulmonary	Oaxaca	M/7 yrs
2002	C1262	■■■■■■■■■■■■■■□■■■■■□□□□■■■■□■■□□□□□□□■□■■■	777767607540131	LAM4	Orphan	Pansusceptible	biopsy	Extra Pulmonary (Abdominal)	Edo De México	M/7 yrs
2002	C1365	■■□■■■■■■■■■■■□■■■■■□□□□■■□■■■■□□□□□□■■■■■■	677767606740371	LAM8	Orphan	Pansusceptible	sputum	Pulmonary	Jalisco	F/18 yrs
2002	C1677	■■□■□□□□□□□□□■■□■■■■■■■■■■■■■■■■■■■■■■■□□■□	640033777777710		Orphan	Pansusceptible	CSF	Pulmonar(Pleural)	Guerrero	M/14 yrs
2002	C1842	■■■□■■□□■□□□■□□■□□■■□□□□■■□■■■■■□□■■■■■■■■■	731044606763771	UNK	Orphan	Pansusceptible	sputum	Pulmonary	Jalisco	F/17 yrs
2002	C521	■■■■■■■■■■■■■■■■■■■■□□□□□■□□□□□□□□■■■■■■■■■	777777602003771		Orphan	Pansusceptible	sputum	Pulmonary	Edo De México	F/18 yrs
2002	C672	■□□□□■□□□□■□□■□□■□□□□□□□□□□□□□□□□□□□□□□■■■	410222000000031		Orphan	Pansusceptible	sputum	Extra Pulmonary (Mliliary)	Hidalgo	F/16 yrs
2002	C615	■■■■■■□□□□■■■□□■■□■■□□■□■■■■■■■□□□□□□■■■■■■	770346627740371	UNK	Orphan	Resistant=I, Rif, Et, Pz	sputum	Pulmonary	Durango	M/11 yrs
2002	C890	■■■■■■■■■□■■■□□□□□□□□□□□■□□□□□□■□□□□■■■■■■■	777340004020771	H1	Orphan	Resistant=I, Rif	sputum	Pulmonary	Jalisco	F/15 yrs
2002	C111	■■■□□■□□■□□□□■□□■□□□■□□□□■■□■■■■■□□□□■■■■■■	711022103370371		Orphan	Resistant=Streptomycin	gastric lavage	Extra Pulmonary (Meninges)	Baja California	F/1 yrs
2002	C1519	■■□■■■■■□■■■■■■□■■■■■■■■■■■■■■■■■■■■■■■□□■□	676773777777710		Orphan	Resistant= I	sputum	Pulmonary	Tamaulipas	M/ 18 yrs
2002	C164	■■■■■■■■■□■■■■□■■■■■□□■□■■□■■■■■□□□□■■■■■■■	777367626760771	T1	Orphan	Resistant= I, Resistant= Pz	biopsy	Extra Pulmonary (Systemic)	Baja California	F/1 yrs
2002	C855	■□□■■■■■■■■■■■■■■■■□□■■■■■■■□□□□□□■■■■■□□□■	477777477403701		Orphan	Resistant= I, Et, Pz	sputum	Pulmonary	Guerrero	F/17 yrs
2003	C1261	■■■□□□□□□□□□■■■■■□■■■■■■■■■■■■■□□□□□□■□■■■■	700076777740271	X3	Orphan	Pansusceptible	sputum	Extra Pulmonary (Lymph nodules)	Chiapas	M/11 yrs
2003	C1283	■■■■■■■■■■■■■■■■■■■■□□□□■■■■□■■■□□□□■□■□■■■	777777607560531	LAM6	Orphan	Pansusceptible	biopsy	Pulmonary	Queretaro	M/3 yrs
2003	C54	■■□■■■■□□□■■■■□□■■■■■■■■■■■■■■■■■■■□□□□□□□□	674363777776000	BOVIS	Orphan	Pansusceptible	sputum	Pulmonary	Sinaloa	F/14 yrs
2003	C655	■■■■■■■■■□■■■■□■■■■■□□■□□■■■□■■□□□□□■■■■■■■	777367623540771	T1	Orphan	Pansusceptible	sputum	Extra Pulmonary (Milliary)	Guanajuato	F/2 yrs
2003	C765	■□■□□■■■■□■■■■□■■■■■□□■■■■■■■■■■□□□□■■■■■■■	517367637760771	T1	Orphan	Pansusceptible	sputum	Pulmonary	Jalisco	F/17 yrs

* SITs followed by an asterisk indicate "newly created shared-type" after match with another orphan in the database, or due to 2 or more strains belonging to a new pattern in this study

Orphan patterns are highlighted in blue

** Drug resistance (abbreviations): S, Strepromycin; I, Isoniazid; Rif, Rifampin; Et, Ethambutol; Pz, Pyrazinamide
